# An in-depth look at shallow-water walking: the mechanical determinants of the energy metabolic cost of shallow water walking in humans

**DOI:** 10.1007/s00424-025-03130-3

**Published:** 2025-12-12

**Authors:** André Ivaniski-Mello, Alberto Enrico Minetti, Flávia Gomes Martinez, Leonardo Alexandre Peyré-Tartaruga

**Affiliations:** 1 LaBiodin Biodynamics Laboratory, Universidade Federal do Rio Grande do Sul, Porto Alegre, Brazil; 2 Laboratory of Physiomechanics of Locomotion, Department of Pathophysiology and Transplantation, Physiology Division, University of Milan, Via Mangiagalli 32, Milan, 20133 Italy; 3 Human Locomotion Laboratory (LocoLab), Department of Public Health, Experimental and Forensic Medicine, University of Pavia, Via Forlanini 2, Pavia, 27100 Italy; 4 Departamento de Biofísica, Facultad de Medicina, Universidad de la República, Montevideo, Uruguay

**Keywords:** Locomotion, Energy cost, Metabolic power, Aquatic walking, Water immersion, Swimming

## Abstract

Human locomotion in water involves unique forces (buoyancy, drag) influencing metabolic cost. However, a validated model integrating these forces to predict the cost of transport (COT) during shallow water walking (SWW) is lacking, and energetic optimization strategies remain unclear compared to terrestrial gaits. We measured the COT in nine healthy men during SWW across four immersion depths (knee to xiphoid) and four walking speeds (0.2–0.8 m/s). We developed and validated a physiomechanical model based on the mechanical work done against buoyancy-affected body weight and hydrodynamic drag. Using this model, we compared the energetics of SWW with swimming and dry land walking (including hypogravity conditions) and analyzed self-selected walking speeds. The minimum COT occurred at hip immersion depth (0.2 m/s), rather than at intermediate speeds, with the J-shaped relationship observed only at knee immersion depth. Metabolic power, in contrast, remained relatively constant during self-selected walking across immersion depths. An immersion depth threshold near the center of mass emerged, above which swimming becomes more economical than SWW. Our physiomechanical model accurately predicted the measured COT. The interplay between buoyancy and drag dictates SWW energetics, shifting optimization away from intermediate speeds common on dry land. From a physiological perspective, these findings quantify the energetic consequences of human locomotor adaptation to the unique mechanical challenges posed by aquatic environments. Furthermore, identifying an immersion depth threshold influencing the economical choice between walking and swimming provides new insights into human aquatic locomotor adaptations.

**Supplementary Information:**

The online version contains supplementary material available at 10.1007/s00424-025-03130-3.

## Introduction

Gait selection during locomotion is often driven by the optimization of physiomechanical parameters, particularly of metabolic energy consumption [1]. Reducing the energy cost of movement confers a substantial evolutionary advantage, preserving resources for other vital tasks such as reproduction, foraging, or predator evasion [2]. This principle is clearly demonstrated in human dry land walking, where the cost of transport (COT) – defined as metabolic energy expenditure normalized by body mass and distance traveled (J/kg/m) – typically exhibits a minimum value at intermediate locomotion speeds [35]. Because the total metabolic energy expenditure depends on the work done against environmental resistances, such as aerodynamic drag [34], the specific environment shapes the COT of human locomotion.

Compared to terrestrial locomotion, movement in water introduces two crucial forces in addition to gravity: buoyancy and drag forces. Buoyancy acts as a hydrostatic vertical force opposing gravity, proportional to the weight of the displaced fluid. This effect reduces apparent body weight [14], creating a simulated hypogravity environment (g < 1.0). Drag, conversely, is a hydrodynamic force that resists displacement through the fluid, acting opposite to the body’s velocity. While drag comprises pressure, frictional, and wave components [37], pressure drag is considered the most critical factor during shallow water walking (SWW) [28]. Despite humans being relatively ill-adapted for aquatic movement, [36], numerous physical, sports and leisure activities are commonly performed in water [13, 39].

Human move through aquatic environments primarily through SWW and swimming. SWW represents an adaptation of terrestrial walking, a behavior also been observed in other *Hominidae* [21], whereas swimming, although the primary mode of locomotion for aquatic species [22], is less mechanically natural for humans. These two gaits differ fundamentally in body orientation and ground interaction. SWW maintains an upright posture, similar to dry land walking, meaning the foot cyclically contacts the ground, momentarily bringing the distal limb velocity to zero [35]. In contrast, swimming involves a predominantly horizontal body position, which alters the frontal area exposed to drag, and relies on fluid gliding without ground contact [37]. Consequently, as with terrestrial locomotion, ground-based gaits like SWW typically achieve lower speeds compared to fluid-gliding gaits like swimming [22].

The mechanical distinctions between SWW (upright posture, ground contact) and swimming (horizontal gliding) directly influence the work required against buoyancy and drag, and thus the overall energetic cost. While previous studies have characterized various physiological and biomechanical responses during SWW [16], and sophisticated models exist for swimming energetics [39], a validated physiomechanical model explaining the SWW metabolic COT by integrating the simultaneous effects of buoyancy and drag has been lacking. SWW is generally more energetically expensive than dry land walking at same speed [16]. However, this relationship may reverse at deeper immersions (above the waist), where the assistive effect of buoyancy potentially overcomes the resistive effect of drag, leading to a net reduction in energy expenditure compared to dry land walking [12]. Developing a model that explicitly links the mechanical work done against aquatic forces to the metabolic cost can therefore provide crucial insights into human locomotor adaptability in water and reveal potential energy optimization strategies.

To develop a comprehensive understanding of human aquatic locomotion, this study aimed to: (1) determine the COT and quantify the buoyancy and drag forces during SWW in healthy men across four immersion depths and four walking speeds; (2) develop and validate a physiomechanical model incorporating these aquatic forces to estimate the SWW COT; and (3) compare the COT during SWW with that of human swimming to better understand natural human aquatic locomotion strategies. Based on these aims, we hypothesized that: H1) the SWW COT would be directly related to the interplay between buoyancy and drag forces; H2) the SWW COT would exhibit a minimum value at intermediate speeds; and H3) SWW would be metabolically more expensive than human swimming.

## Materials and methods

### Participants and ethics

Nine men (mean ± standard deviation (SD): 28 ± 8 years, 77.1 ± 9.8 kg, 1.78 ± 0.04 m) were analyzed while walking in shallow water. All participants were healthy and had no neurological or musculoskeletal conditions that could impair their walking ability. The study was approved by the ethics committee of Universidade Federal do Rio Grande do Sul, Brazil (project number 37928). All participants were informed of the potential risks associated with the experimental protocol and provided written informed consent. The project was also registered at the Open Society Foundations (doi: 10.17605/OSF.IO/JFYXN).

### Data collection

Data collection was conducted over two non-consecutive days, with at least one week between sessions. Participants were instructed to walk at four immersion depths (knee, hip, umbilicus, xiphoid) at four fixed speeds (0.2, 0.4, 0.6, 0.8 m/s), as well as at a self-selected comfortable speed for each immersion depth. The order of the immersion depths and walking speeds was randomized. Participants completed trials at two immersion depths on each day, thus covering all four depths across two sessions. Anthropometric measurements of the lower limbs and trunk, including segment lengths and circumferences, were also collected.

**Fig. 1:**
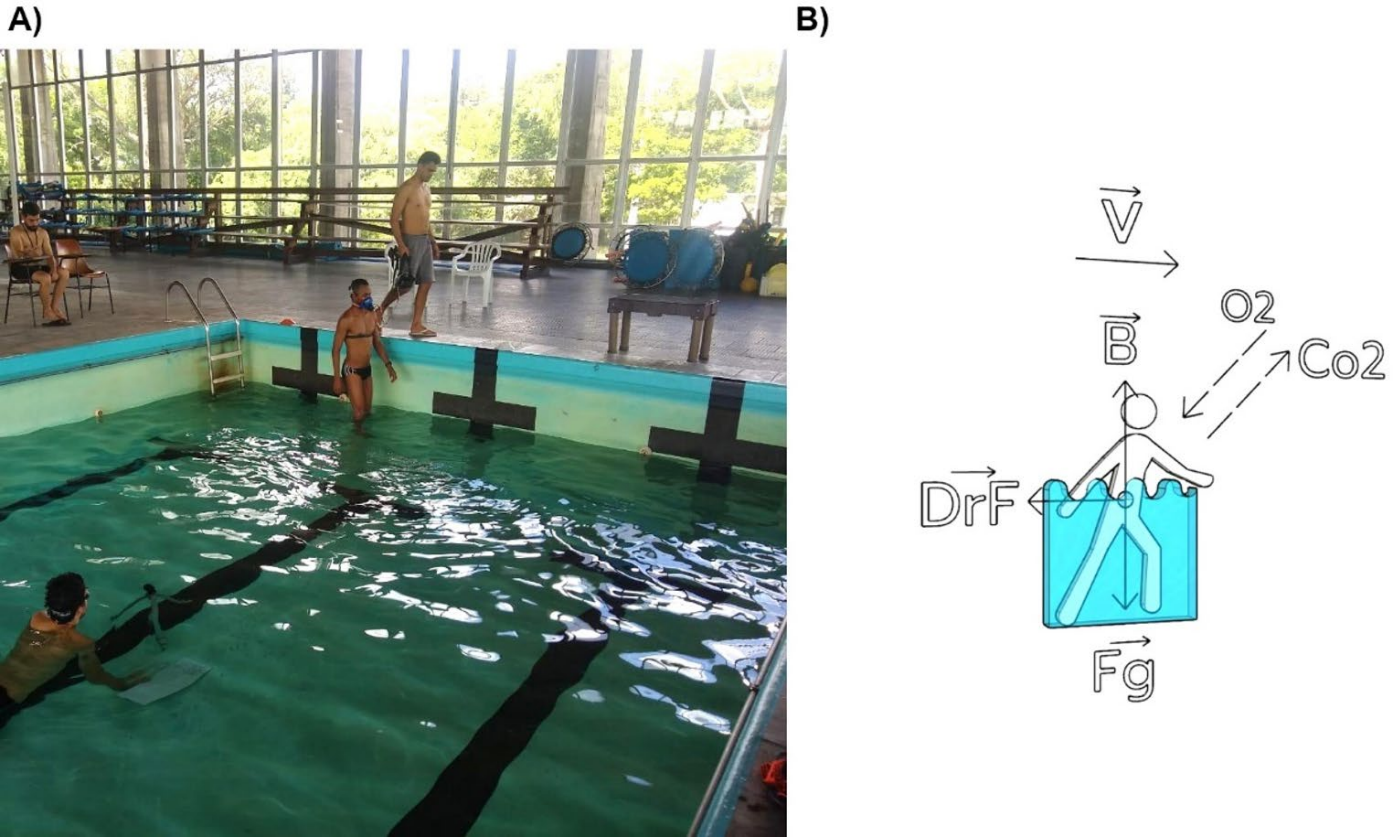
(**A**) Experimental setup for shallow water walking trials. The participants wore a portable gas analysis system to measure metabolic cost while kinematic data were collected simultaneously by a waterproof camera. The procedure was repeated at four immersion depths (knee, hip, umbilicus, xiphoid) and four different walking speeds (0.2, 0.4, 0.6, 0.8 m/s). (**B**) Physiomechanical model of human shallow water walking. B: buoyancy force. CO_2_: carbon dioxide. D: drag force. Fg: gravitational force. O_2_: oxygen. v: walking velocity

The walking tests were conducted in 16 × 6 m pool with a depth of 1.4 m. To reach the desired immersion depth for each condition, water was removed from the pool when necessary. The tests took place in a climate-controlled environment with thermoneutral water maintained at 31–32 °C (Fig. 1A). Because the pool depth was fixed, the immersion depths are presented in metric units for each condition, along with the corresponding mean ± SD (in percentage of participants’ stature): knee, 0.5 m (28 ± 1%); hip, 0.85 m (48 ± 1%); umbilicus, 1.12 m (63 ± 1%); xiphoid, 1.3 m (73 ± 2%).

Walking speed was controlled using a timed auditory cue and marked positions every 2.5 m along the pool edge. During fixed-speed conditions, participants were instructed to walk from one marker to the next in synchronization with the auditory cues. In contrast, during the self-selected comfortable speed condition, participants chose their own walking pace.

The walking tests were conducted along a back-and-forth path. Participants walked 5 m and then turned 180º at each endpoint. To evaluate whether this turning pattern affected energy expenditure, a pilot study was conducted (*n* = 2, 25 ± 0 years, 84.5 ± 4.9 kg, 1.80 ± 0.08 m; mean ± SD) in a larger pool, where participants could walk 10 m before turning. Comparison of the two conditions revealed a mean difference of only 2.2 ± 1.7% higher COT for the 5 m path compared with the 10 m path.

Each walking speed trial lasted 5 min, followed by a 3 − 5-minute rest interval. The next walking condition began only after participant’s heart rate (monitor Polar FT1, Polar, Kempele, Finland) and rating of perceived exertion (6–20 Borg’s scale [6]) had returned to baseline levels. To account for the influence of water immersion on cardiovascular recovery, a longer rest interval was provided between trials at different immersion depths. Specifically, when participants first walked at a deeper immersion depth (e.g. xiphoid), a 15-minute interval was given before initiating the subsequent trial at a shallower depth (e.g. umbilicus) [11].

Kinematic data were collected using a waterproof GoPro Hero 5 (GoPro Inc., San Matea, USA) at 60 Hz. The camera was positioned 4 m from the participant during the walking tests, with the lens aligned perpendicular to the sagittal plane of motion. It was placed at a height of 0.6 m for the hip, umbilicus, and xiphoid immersion depths, and 0.5 m for the knee immersion depth, measured from the pool floor. To calibrate the movement area to metric scale, a rectangular calibrator frame measuring 2.1 × 1.6 m, with 0.10 m distances between markers, was used. Anatomical landmarks on the right side of the body were marked with waterproof ink at the fifth metatarsal, calcaneus, lateral malleolus, lateral femoral epicondyle, greater trochanter, and the lateral projections of the umbilicus and xiphoid process.

The oxygen consumption and carbon dioxide production were collected using a K5 portable gas analysis system (COSMED, Rome, Italy) in breath-by-breath mode, calibrated according to the manufacturer’s instructions. At each immersion depth, respiratory gas exchange at rest (orthostatic posture) was recorded for 7 min. During the walking tests, respiratory gases were collected throughout the 5- minute walking trials; however, only the data from the final 2 min were used for analysis.

### Data analysis

#### Shallow water walking

The SWW videos were imported into SkillSpector software (v. 1.2.3, Video4Coach, Copenhagen, Denmark), where the anatomical landmarks were manually digitized. Five strides per participant were analyzed for each speed condition, totaling 700 strides. The position data of each anatomical marker were exported and processed in a custom MATLAB routine (2020a, Mathworks Inc., Massachusetts, USA). Details of the routine are available online (https://github.com/andreivaniskimello/Gait-Analysis). The kinematic data were filtered using a 2nd order low-pass Butterworth filter (4–5 Hz). Walking speed (m/s), the stride length (m), the angular position and angular speed of each segment were then calculated. These spatiotemporal and angular variables were subsequently used to estimate the drag force during stride, as described in the “Physiomechanical model of shallow water walking” section.

Energy expenditure was estimated by indirect calorimetry from the K5 data [17, 32]. Oxygen and carbon dioxide measurements were used to calculate the gross metabolic power (J/kg/min)- for each condition (at-rest and walking). Net metabolic power (J/kg/min) was obtained by subtracting the at-rest gross metabolic power at the corresponding immersion depth from the walking gross metabolic power. The COT (J/kg/m) was calculated by dividing net metabolic power by the walking speed. Participants were assumed to rely primarily on aerobic metabolism at all walking speeds, with the mean ± SD respiratory exchange ratio for each speed as follows: 0.2 m/s, 0.84 ± 0.03; 0.4 m/s, 0.80 ± 0.02; 0.6 m/s, 0.79 ± 0.01; 0.08 m/s, 0.87 ± 0.05. In addition, at-rest gross metabolic power values were used to compare the heat transfer between the skin and water across immersion depths.

#### Swimming

For the human swimming data, the metabolic cost of transport was estimated using the equation proposed by Capelli et al. [7] for front crawl swimming. The front crawl stroke was selected as a reference because it exhibits greater metabolic efficiency than both the backstroke and breaststroke across a wide range of speeds [15, 39]. To evaluate human aquatic locomotion from an energetic and evolutionary perspective, data from the most energetically economical swimming style were therefore adopted. Although the equation by Capelli et al. [7] was originally derived for front crawl above 0.96 m/s, it was employed in the present study to allow comparison with SWW, while acknowledging the inherent limitations of extrapolating it to lower speed range analyzed. The self-selected swimming speed was defined as the speed maintained during a two-hour front crawl swimming test [5].

#### Physiomechanical model of shallow water walking

The physiomechanical model of SWW (Fig. 1B) was developed using the collected physiological data - the COT - and the estimated kinetic data -the total drag force during the stride cycle (DrF) and the mean vertical ground reaction force during the stride cycle (GRF_V_).

The drag force (N) was estimated using the mathematical model of Orselli and Duarte [29] incorporating anthropometric and kinematic data (Supplementary Material 1). Each lower limb and trunk segment was modeled as a conic frustum based on segment lengths and circumferences. Kinematic data were used to calculate the linear velocity and angular position of each segment. The angular position was then used to determine the frontal projected area of the segment in the frontal plane. Strip theory was applied to estimate the drag force: each segment was divided into multiple thin strips, and the drag force was calculated for each strip at every time point. The total drag force was calculated by summing the forces across all strips during both the contact and swing phases, and the combined value of these two phases (DrF) was used for statistical analyses and in the physiomechanical model. A turbulent flow regime around the body segments was assumed, supported by the high Reynolds numbers typically observed during human locomotion in water [28]. Under these conditions, pressure forces dominate over frictional forces, making a pressure drag–based model [30] appropriate for shallow water walking.

To evaluate the effect of buoyancy force in each walking condition, the GRF_V_ (N) was estimated based on the apparent body weight at each immersion depth (Supplementary Material 1). Considering that GRF_V_ over the stride cycle equals the participant’s body weight [25], we calculated the apparent body weight (expressed as percentage of dry land weight) for each immersion depth, accounting for the weight-bearing reduction due to buoyancy force [20]. The apparent body weight was then used as the stride GRF_V_.

To compare our SWW data with the dry land hypogravity walking [31], gravity acceleration (g) was converted into corresponding immersion depth (m) using literature data on water-immersed apparent weight reduction [20]. The apparent weight reduction at each analyzed immersion depth was calculated for each anatomical landmark [20], and treated as a simulated hypogravity condition, expressed as a fraction of Earth’s gravity (1 g). This allowed us to determine the equivalent gravitational acceleration foreach immersion depth. The analyzed immersion depths correspond to gravity equivalents of 0.88 g for the knee, 0.58 g for the hip, 0.48 g for the umbilicus (slightly higher than Mars’s gravity of 0.38 g), and 0.33 g for the xiphoid (nearly twice that of the Moon’s gravity, 0.17 g).

#### Predicted cost of transport during shallow water walking (COT_predicted_)

To test the validity of our physiomechanical model of SWW, we computed the predicted cost of transport (COT_predicted_) based on the mechanical work required to support the body mass vertically and to move the body horizontally against resistive forces during the stride cycle. Total mechanical work was first estimated by summing the horizontal and vertical components. The COT_predicted_ associated with this total mechanical work was then calculated using the efficiency definition [33]. Finally, the predicted COT_predicted_ was compared with the experimentally measured COT for each SWW condition. Further details on the computation of COT_predicted_ are provided below.

The total mechanical work *(*W_Aqua_, J) was partitioned into its horizontal and vertical components. The horizontal work (W_D_, J) represents the work performed by the body to overcome the hydrodynamic drag force over a single stride. The W_D_ was calculated for each i-th stride as:

 

1 $$\:{W}_{{D}_{i}}={DrF}_{i}*{SL}_{i}\:$$

where DrF_i_ (N) is the total drag force during the stride and SL_i_ (m) is the stride length.

The vertical work (W_v,_ J) represents the work done to lift the body’s center of mass (COM) against the apparent body weight during each stride. First, the vertical displacement of the COM for a single contact phase from the i-th stride (COM_Vert_) - in m - was first estimated using the model proposed by Cavagna [38]. For this, the lower limb length (L_leg_, m) and the forward displacement during single contact phase (L_sc_, m) were used:

 

2 $$\:{CO{M}_{Vert}}_{i}={L}_{leg}\left(1-cos\left(0.5*{{L}_{sc}}_{i}/{L}_{leg}\right)\right)\:$$

The vertical work for the i-th stride (W_v_, J) was then determined by the work required to lift the COM against the apparent body weight, represented by the *GRF*_*v*_, during each stride (two contact phases):

 

3 $$\:{Wv}_{i}={{GRF}_{v}}_{i}*\left({CO{M}_{Vert}}_{i}*2\right)\:$$

By using the GRF_v_, this calculation explicitly accounts for the supportive, weight-bearing effect of buoyancy.

Finally, the W_Aqua_ (Eq. 4) of each i stride was calculated as the sum of the horizontal work against the drag force (W_D_) and the vertical work done to lift the center of mass against the apparent body weight (W_v_), which accounts for the effect of buoyancy

 

4 $$\:{WAqua}_{i}={WD}_{i}+{Wv}_{i}$$

The COT_predicted_ (J/kg/m) of SWW in each *i* stride was then estimated (Eq. 5) by dividing the W_Aqua_ by the body mass (kg), the stride length (SL, m), and an assumed muscle efficiency of 0.25. We adopted a muscle efficiency of 0.25 by considering the predominance of concentric muscle actions during movements in water-immersed conditions [4, 33]. This assumption is supported by evidence from analogous aquatic movements, such as jumping, where both buoyancy and hydrodynamic drag significantly reduce the eccentric “braking” work required compared to land-based movements [23].

 

5 $$\:{{COT}_{predicted}}_{i}=\frac{{WAqua}_{i}}{\left({SL}_{i}*body\:mass*0.25\right)}$$

The mean COT_predicted_ along all strides for each condition of immersion depth and speed was computed for each participant, and this mean value was used for the subsequent analyses.

### Statistical analyses

The results are presented as mean, standard deviation, and 95% confidence interval (95% CI). The statistical analyses were performed using custom Python scripts and the Statistical Package for Social Sciences (SPSS, v.26, Chicago, Illinois, USA), with α = 0.05 for all analyses.

#### Preliminary analyses

Simple t-tests were used to verify that participants achieved the target walking speed in each fixed-speed condition. To confirm that our calculation of net metabolic power effectively accounted for baseline metabolic costs unrelated to walking (e.g., thermoregulation), a Generalized Linear Mixed Model (GLMM) compared the gross metabolic power during the resting condition across the four immersion depths, followed by a Bonferroni post hoc test.

#### Analysis of immersion depth and walking speed effects (hypothesis 1 -H1)

To test our first hypothesis - that the SWW COT response is related to the interplay between buoyancy and drag - a GLMM was used. This model assessed the main effects of immersion depth (knee, hip, umbilicus, xiphoid) and walking speed (0.2, 0.4, 0.6, 0.8), as well their interaction (immersion depth*walking speed) on the dependent variables: COT, DrF and GRF_v_. Bonferroni post hoc tests were used for pairwise comparisons where appropriate. Eta-squared (η^2^) effect sizes were calculated for all main effects and interaction. Furthermore, Pearson correlation tests were performed to quantify the association between COT and the kinetic parameters (DrF and GRF_v_), with the Pearson correlation coefficient (r) reported.

#### Polynomial modeling and optimization (hypothesis 2- H2)

To investigate potential optimization and test our second hypothesis - that SWW COT would exhibit a minimum value at intermediate speeds - second-degree polynomial regression models were employed. First, a 3D surface plot of COT as a function of both immersion depth and walking was generated using a polynomial fit to the experimental data, allowing for the identification of overall minimum and maximum COT values within the tested range. Second, to specifically test for a minimum COT at intermediate speeds for each immersion depth (analogous to the U-shaped curve in dry land walking), the experimental SWW COT data were fitted with separate second-degree polynomial regression curves for each immersion depth, plotting COT as a function of speed. The shape of these curves directly addresses H2. The 3D surface for dry land hypogravity condition was generated based on the equation by Pavei & Minetti [31]. The COT at the self-selected comfortable speed for each immersion depth was estimated using the 3D polynomial, and the corresponding metabolic power (W/kg) was calculated.

#### Physiomechanical model validation

The validity and robustness of the developed physiomechanical model (predicting COT_predicted_) were assessed through several methods. First, the ratio between COT_predicted_ and the measured COT was calculated for each condition, and the overall coefficient of determination R^2^, with its associated p-value) between predicted and measured values was computed. Second, the model’s predictive robustness was tested using leave-one-out cross-validation (LOOCV) to determine the cross-validated R^2^. Third, the stability of the R^2^ correlation was evaluated using a bootstrapping analysis (1,000 iterations) to generate a 95% CI. Finally, a sensitivity analysis quantified the model’s response to ± 10% variations in key input parameters (muscle efficiency, body mass, walking speed, stride length), including a quadratic adjustment for drag during the speed variation analysis. At each input parameter modification, the COT_predicted_ was recalculated, and the resulting mean percentage change was determined to quantify the model’s sensitivity.

#### Comparative analyses (hypothesis 3 - H3)

To test our third hypothesis - that SWW would be metabolically more expensive than swimming - and to compare SWW with dry land walking, we calculated iso-cost points. The polynomial regression curves of COT versus speed for each SWW immersion depth were compared against the established curves for human swimming [7] and dry land walking [3]. The intersection points identify the speeds at which SWW has a similar COT to swimming or dry land walking, directly addressing H3 by showing the conditions under which one mode is more economical than another.

## Results

### Shallow water walking

The individual dataset is available online (doi: 10.6084/m9.figshare.13221485) and in Supplementary Material 2. Participants were able to achieve the target walking speeds, except at 0.8 m/s across all immersion depths (knee: 0.72 ± 0.04 m/s, *p* = 0.002; hip: 0.73 ± 0.20 m/s, *p* < 0.001; umbilicus 0.76 ± 0.04 m/s, *p* = 0.032; xiphoid 0.64 ± 0.03 m/s, *p* = 0.008).

Table 1 presents the mean values, 95% CI, and statistical comparison results for COT, DrF, and GRF_v_ at each immersion depth and walking speed. The COT increased with greater immersion depth (*p* < 0.001; η^2^ = 0.33) and walking speed (*p* < 0.001; η^2^ = 0.60), with a significant depth*speed interaction (*p* < 0.001; η^2^ = 0.43). Only at 0.2 m/s did the COT remain similar across all immersion depths. The overall minimum COT, considering all immersion depths and walking speeds, occurred at hip immersion depth at 0.2 m/s. Furthermore, gross metabolic power at rest was similar across immersion depths (*p* = 0.06; η^2^ = 0.22), indicating that the metabolic cost associate with heat transfer between skin and water was comparable across immersion depths.

The DrF increased with both greater immersion depth (*p* < 0.001; η^2^ = 0.58) and walking speed (*p* < 0.001; η^2^ = 0.88), with a significant depth*speed interaction (*p* = 0.001; η^2^ = 0.20). DrF values at the umbilicus and xiphoid immersion depths were similar, and at xiphoid immersion depth DrF did not change between 0.6 and 0.8 m/s walking speed. In contrast, GRF_V_ decreased with increasing immersion depth (*p* < 0.001; η^2^ = 1.00).

### Physiomechanical model of shallow water walking

**Fig. 2:**
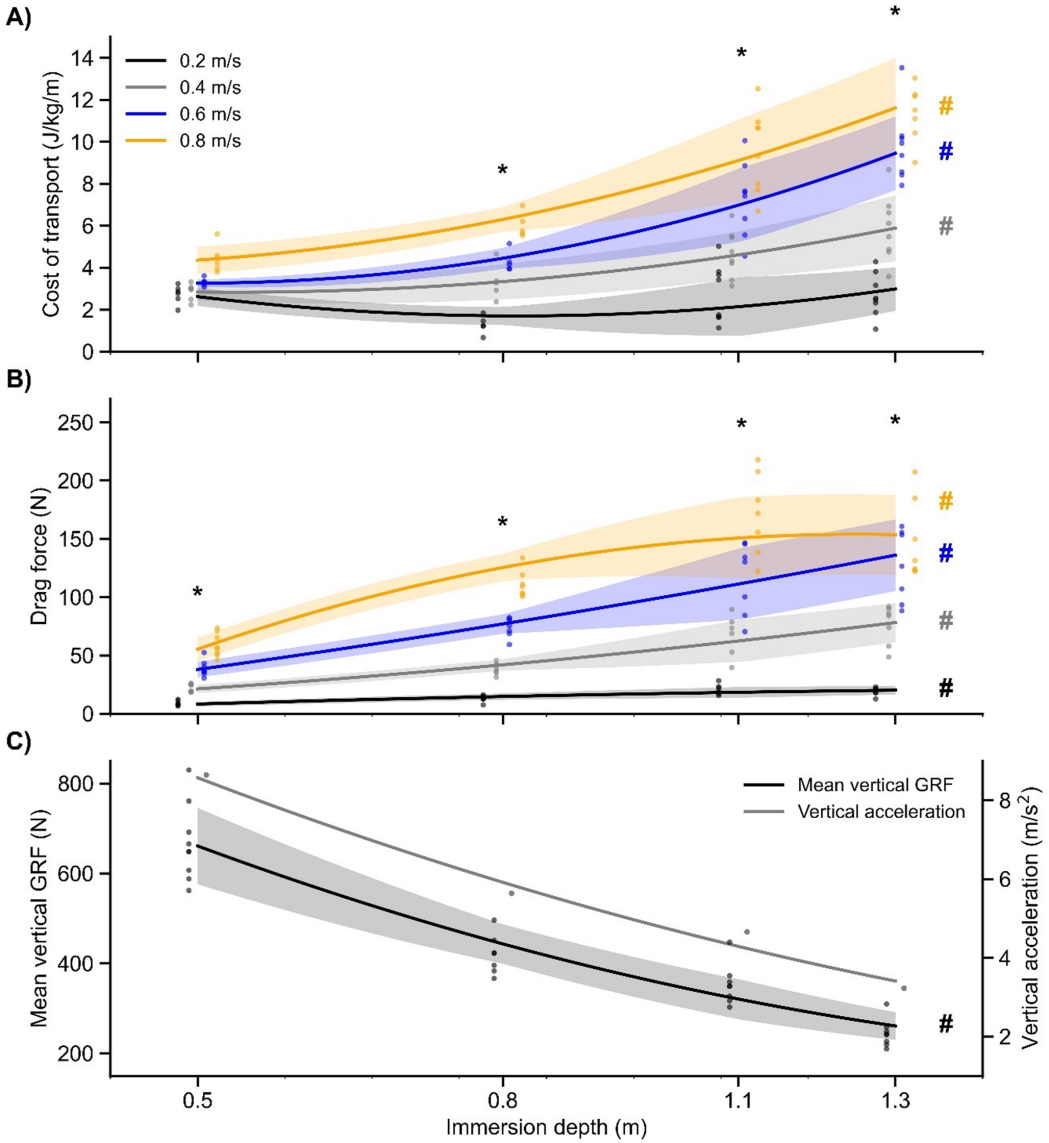
Physiomechanical responses during shallow water walking at different immersion depths and walking speeds. **A** Cost of transport (J/kg/m), (**B**) drag force (N), and (**C**) mean vertical ground reaction force (GRF, N) and vertical acceleration (m/s^2^) plotted against immersion depth (m) during shallow water walking. Solid lines represent 2nd-order polynomial fits to the mean data for each walking speed condition (colors indicated in the legend). Shaded areas represent the mean ± 1 standard deviation of the individual data, interpolated along the polynomial fit. In panel C, GRF and acceleration data are plotted against the left and right y-axes, respectively. Significance markers indicate differences (*p* < 0.05) based on post-hoc tests (detailed in Table 1): * denotes significant differences across walking speeds within a specific immersion depth; # denotes significant differences across immersion depths within a specific walking speed

Figure 2 illustrates the responses of COT, DrF, and GRF_V_ at different immersion depths for each walking speed. DrF was positively correlated with COT (*r* = 0.88, *p* < 0.001), whereas GRF_V_ was negatively correlated with COT (*r* = −0.39, *p* < 0.001).

**Table 1:** Mean and 95% confidence intervals of the cost of transport, drag force, and mean vertical ground reaction forces during shallow water walking across immersion depths and walking speeds

Variable	Immersion depth/Walking speed	0.2 m/s	0.4 m/s	0.6 m/s	0.8 m/s
Cost of transport (J/kg/m)	**knee**	2.7 (2.3–3.2) ^**A/a**^	2.8 (2.4–3.3) ^**A/a**^	3.3 (3.1–3.5) ^**A/a**^	4.4 (3.8–5.1) ^**A/a**^
	**hip**	1.3 (0.8–1.8) ^**A/a**^	3.3 (2.3–4.4) ^**A, B/a**^	4.3 (3.7–4.9) ^**B/a**^	6.0 (5.3–6.7) ^**C/a**^
	**umbilicus**	2.8 (1.6–3.9) ^**A/a**^	4.7 (3.7–5.6) ^**A/a, b**^	7.2 (5.8–8.7) ^**B/b**^	9.6 (7.9–11.2) ^**C/b**^
	**xiphoid**	2.7 (1.8–3.6) ^**A/a**^	5.9 (4.6–7.2) ^**B/b**^	9.8 (8.3–11.2) ^**C/c**^	12.1 (10.1–14.1) ^**C/b**^
Drag force (N)	**knee**	8.7 (7.0–10.3.0.3) ^**A/a**^	22.0 (19.1–24.9) ^**B/a**^	38.6 (33.0–44.3.0.3) ^**C/a**^	58.7 (50.3–67.1) ^**D/a**^
	**hip**	13.0 (10.4–15.5) ^**A/b**^	38.8 (34.3–43.3) ^**B/b**^	74.0 (66.3–81.7) ^**C/b**^	111.3 (100.5–122.0) ^**D/b**^
	**umbilicus**	20.9 (16.0–25.7.0.7) ^**A/c**^	67.1 (48.1–86.1) ^**B/c**^	115.9 (87.4–144.3.4.3) ^**C/c**^	170.9 (138.5–203.3.5.3) ^**D/c**^
	**xiphoid**	19.0 (15.1–22.8) ^**A/c**^	76.0 (60.3–91.5) ^**B/c**^	126.3(97.9–154.8.9.8) ^**C/c**^	148.9 (117.3–180.5.3.5) ^**C/b, c**^
GRF_V_ * (N)	**knee**	666.7 (642.4–691.0) ^**a**^			
	**hip**	419.6 (405.4–433.7.4.7) ^**b**^			
	**umbilicus**	352.7 (339.3–366.2.3.2) ^**c**^			
	**xiphoid**	244.3 (235.0–253.6.0.6) ^**d**^			

GRF_V_: mean vertical ground reaction force during the stride cycle. Superscript letters indicate the results of post hoc comparisons. Different uppercase letters (A, B, C, D) denote statistically significant differences between speeds. Different lowercase letters (a, b, c, d) denote statistically significant differences between immersion depths. Conditions that share the same letter are not significantly different. *: Apparent body weight comparisons were only made between immersion depths without considering the different walking speeds.

**Fig. 3:**
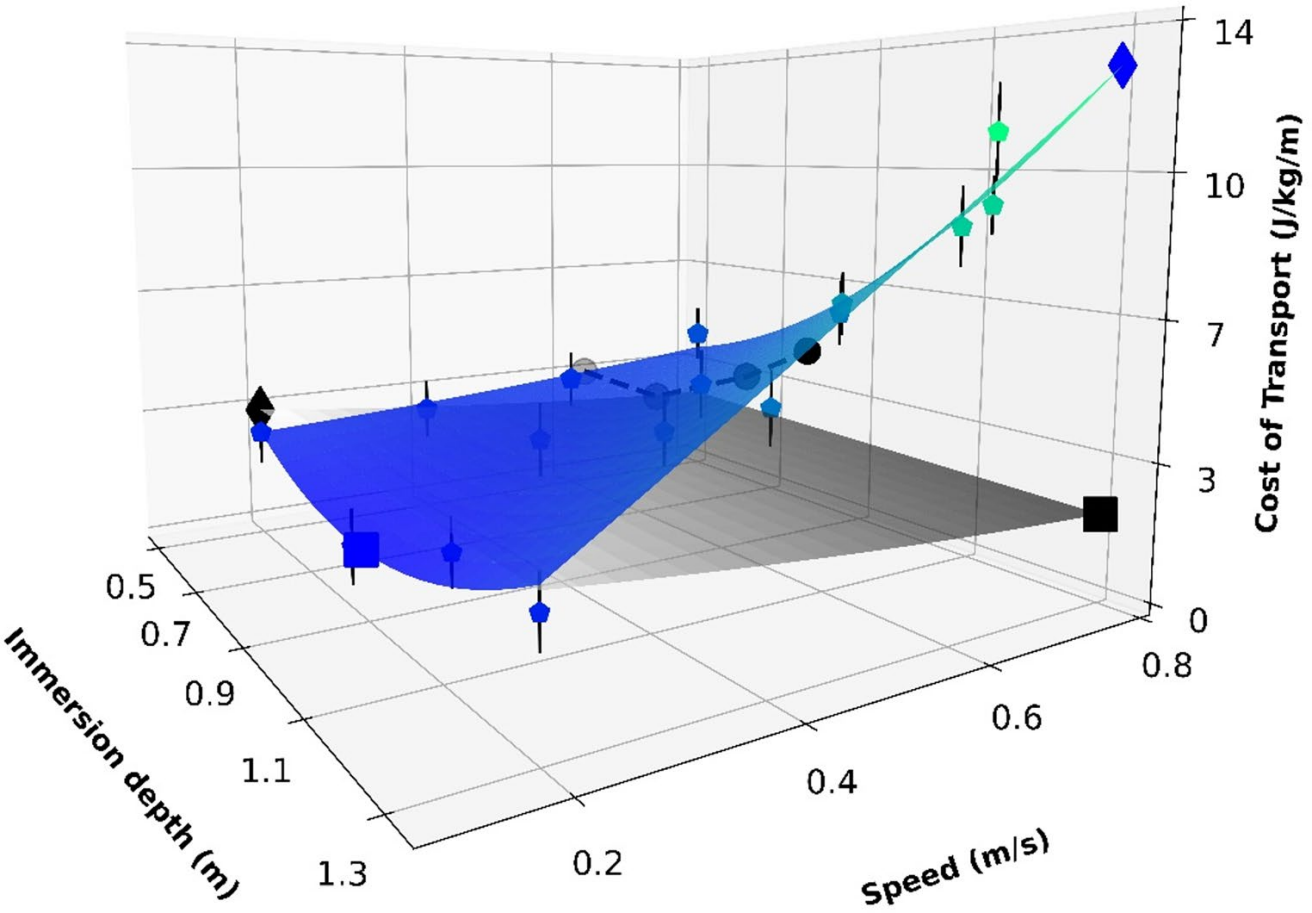
Cost of transport (J/kg/m) during shallow water walking (colored surface and points) and dry land simulated hypogravity walking (grey), as a function of immersion depth (m) and walking speed (m/s). For the dry land condition, the gravitational acceleration (m/s^2^) was converted into immersion depth as detailed in the Methods section. The colored surface represents a 2nd-order polynomial fit to the mean experimental shallow water walking data (R^2^ = 0.98, Eq. 6). Colored scatter points show the mean experimental cost at each condition, with vertical bars indicating the 95% confidence interval. The grey surface represents the predicted cost for dry land walking under simulated hypogravity, based on the model by Pavei & Minetti [31]. Minimum (cube) and maximum (diamond) points of cost of transport are marked for both the shallow water (blue) and dry land (black) surfaces. Black circles indicate the cost at the mean self-selected walking speed for each immersion depth, connected by a dashed line. For detailed statistical comparisons between conditions, please refer to Table 2

The regression of COT during SWW as a function of immersion depth and walking speed was best described by a 2nd -degree polynomial (Eq. 6 and Fig. 3) with an R^2^ of 0.98; the polynomial parameters with 95% CI are provided in Eq. 6. Table 2 presents the minimum and maximum COT values predicted by Eq. 6 for SWW, as well as those predicted for dry land hypogravity walking using the equation from [31]. The corresponding immersion depths and walking speeds at which these minimum and maximum values occurred are also indicated in Table 2. The relationship between immersion depth and equivalent gravitational acceleration is detailed in the Methods Sect. 

6 $$\begin{array}{c}\mathrm{COT}=\left(1.22\:\lbrack-7.1715\::\:9.6177\rbrack\ast{speed}^2\right)\\+\left(9.96\:\lbrack5.3278\::\:4.5990\rbrack\ast\:{depth}^2\right)\\+\left(17.93\:\lbrack12.7915\::\:23.0697\rbrack\ast speed\ast depth\right)\\-\left(8.17\:\left[-18.4388\::\:2.0991\right]\ast speed\right)\\-\left(20.95\:\left[-29.8331:-12.0496\right]\ast depth\right)\:\\+10.52\:\lbrack5.6813:15.3491\rbrack\end{array}$$

where *depth* is the immersion depth (m), and *speed* is the walking speed (m/s).

**Table 2:** Minimum and maximum values of the cost of transport (J/kg/m) and their corresponding immersion depth (m) and walking speed (m/s) derived from surface regression model for shallow water and dry land hypogravity. The cost of transport for dry land hypogravity was estimated using the equation of pavei and Minetti [31]. The correspondence between immersion depth (m) and gravitational acceleration (m/s^2^) for dry land hypogravity walking was determined from standing weight-bearing reduction data reported in the literature [20]

Condition	Surface model	Cost of transport (J/kg/m)	Immersion depth (m)	Walking speed (m/s)
Minimum	**Shallow water**	1.3	0.9	0.2
	**Dry land simulated hypogravity**	2.0	1.3	0.8
Maximum	**Shallow water**	13.0	1.3	0.8
	**Dry land simulated hypogravity**	3.4	0.5	0.2

**Fig. 4:**
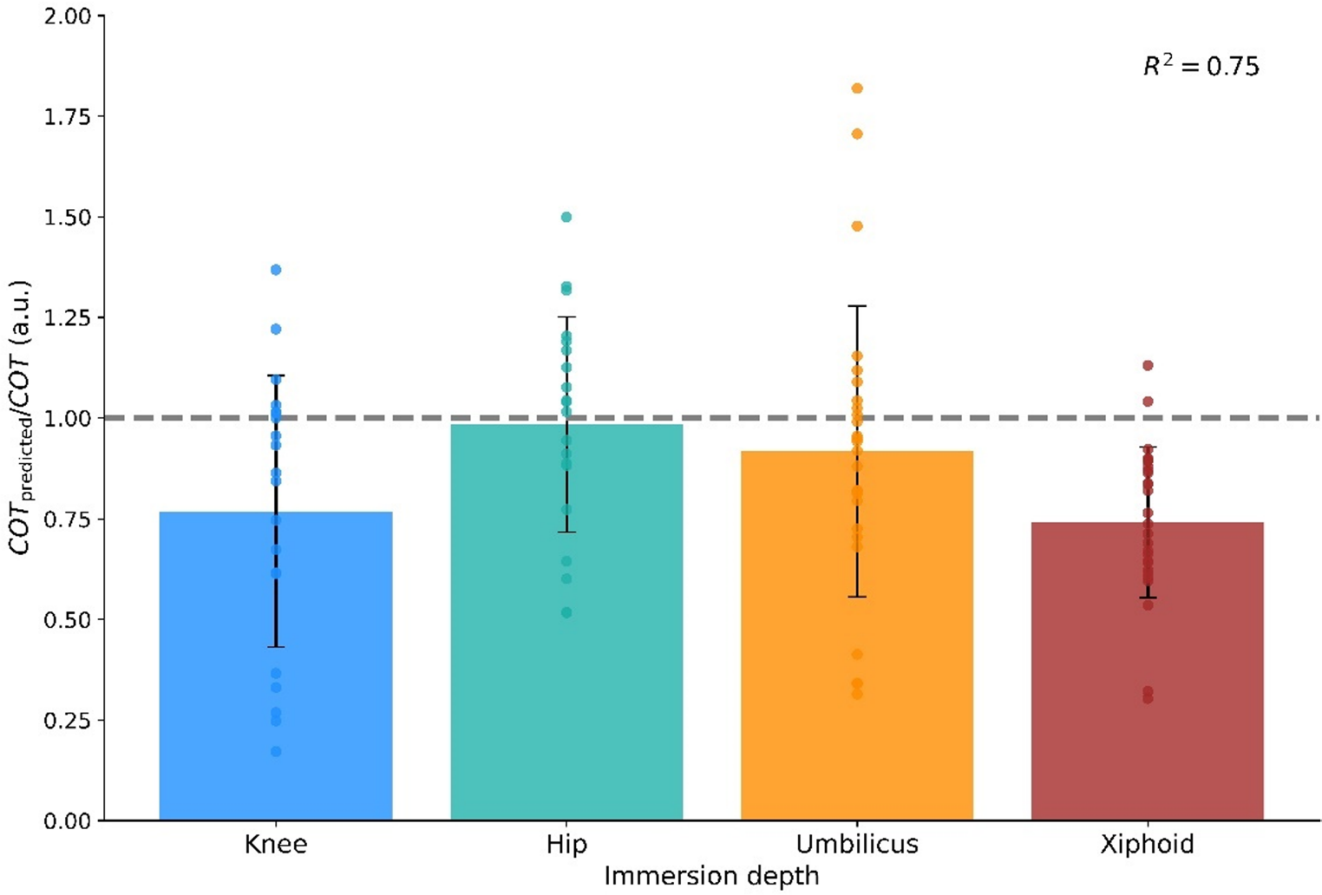
Ratio between the predicted cost of transport (COT_predicted_) and the experimentally measured cost (COT) during shallow water walking at four immersion depths. Each bar represents the mean ratio (± standard deviation) pooled across four walking speeds (0.2, 0.4, 0.6, 0.8 m/s) for each immersion depth (knee: blue, hip: green, umbilicus: orange, xiphoid: brown). Individual participant ratios for each condition are shown as scatter points. The dashed grey line indicates a ratio of 1.0 (perfect agreement). The overall coefficient of determination between predicted and measured COT across all conditions (R^2^ = 0.75) is shown in the top-right corner. Data are presented in arbitrary units (a.u.)

The COT_predicted_ predicted by the physiomechanical model of SWW was similar to the measured experimentally COT across all the immersion depths (Fig. 4). The ratio between the mean values of COT_predicted_ and COT was 0.8 for the knee and xiphoid immersion depths and 1.0 for hip and umbilicus immersion depths, with an overall R^2^ = 0.76 (*p* < 0.001) between COT_predicted_ and COT when all immersion depths were considered together.

Leave-one-out cross-validation yielded a cross-validated R^2^ of 0.75, and bootstrap analysis resulted in R^2^ = 0.70 [95% CI: 0.60–0.79]. Sensitivity analysis indicated that varying muscle efficiency and body mass by ± 10% produced proportional inverse changes in COT_predicted_ of approximately ± 10%. In contrast, varying walking speed by ± 10% induced a highly amplified direct change of approximately ± 20% in COT_predicted_, reflecting the quadratic influence of velocity on drag force. Varying stride length by ± 10% resulted in a dampened inverse change of approximately ± 6–7% in COT_predicted_.

### Shallow water walking vs. swimming and dry land walking

**Fig. 5:**
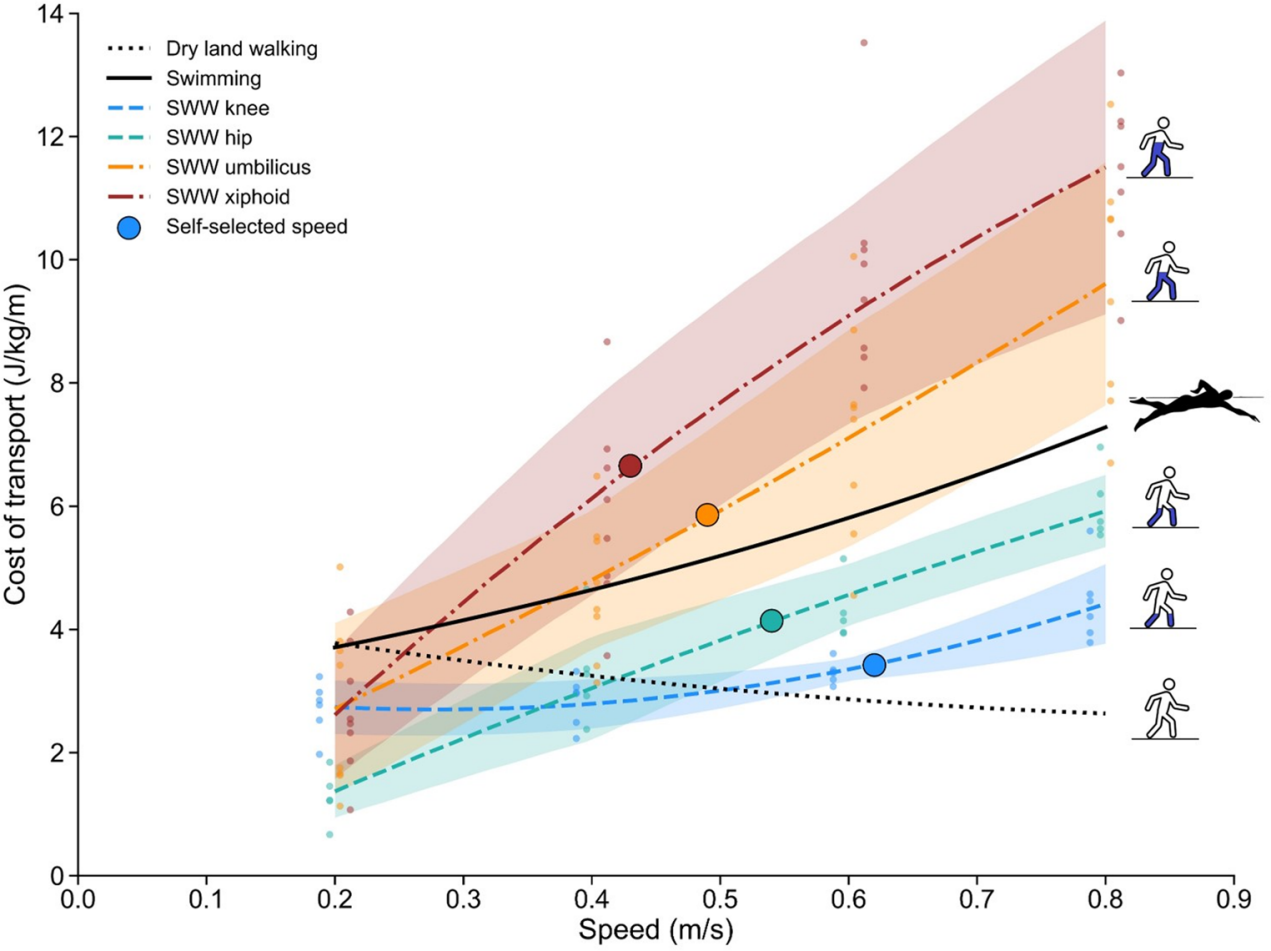
Cost of transport (J/kg/m) as a function of speed (m/s) during different human locomotion modes. Lines represent 2nd-order polynomial fits to mean shallow water walking (SWW) data at four immersion depths (colored lines), mean dry land walking based on Ardigò et al. [3] (dotted black line), and predicted swimming cost based on Capelli et al. [7] (solid black line). Shaded areas around SWW lines indicate mean ± 1 standard deviation. Small colored circles represent individual participant data for SWW conditions. Large colored circles indicate the cost at the mean self-selected speed for each SWW immersion depth. Detailed statistical comparisons are provided in Table 2, and calculated iso-cost points are shown in Table 3

Figure 5 presents the mean COT values for SWW measured at different immersion depths and walking speeds, alongside COT values for swimming. At slow speeds (< 0.36 m/s), SWW exhibit lower COT than swimming across all immersion depths (knee, hip, umbilicus, xiphoid). Additionally, SWW showed lower COT than swimming across the entire speed range at the knee and hip immersion depths. In contrast, at umbilicus and xiphoid immersion depths, SWW exhibited higher COT than swimming at speeds above 0.36 m/s. Notably, SWW only displayed a J-shaped COT-speed relationship at the knee immersion depth, with a minimum COT occurring at the intermediate walking speeds (Fig. 5).

The iso-cost points between SWW and swimming (Table 3) were observed at the umbilicus and xiphoid immersion depths, but not at the knee and hip immersion depths. A transition speed from walking to swimming was observed only at these deeper immersion depths; at shallower depths, COT remained lower for SWW than for swimming. Furthermore, the iso-cost point between SWW and dry land walking occurred at progressively slower speeds with increasing immersion depths.

**Table 3:** Iso-cost points between shallow water walking (SWW) at four immersion depths (knee, hip, umbilicus, xiphoid) with swimming and dry land walking. No metabolic-equivalent speed with swimming was identified at the knee and hip immersion depths, as swimming exhibited higher cost of transport values across all speed ranges

	Swimming		Dry land walking		
Immersion depth of SWW	Cost of transport (J/kg/m)	Speed (m/s)	Cost of transport (J/kg/m)		Speed (m/s)
knee	--	--	3.0	0.49	
hip	--	--	3.2	0.41	
umbilicus	4.5	0.36	3.6	0.27	
xiphoid	4.0	0.26	3.5	0.25	

**Table 4:** Notation of key variables and symbols

Symbol	Description	Units
B	Buoyancy force	N
COM _vert_	Vertical displacement of the center of mass per stride	m
COT	Cost of transport (experimentally measured)	J/kg/m
COT _predicted_	Predicted cost of transport (from the model)	J/kg/m
DrF	Total drag force during the stride	N
Fg	Gravitational force	N
GRF _v_	Mean vertical ground reaction force during the stride cycle	N
L _leg_	Lower limb length	m
LOOCV	Leave-one-out cross-validation	
L _sc_	Forward displacement during single contact phase of a stride	m
MET	Metabolic equivalent of task	
r	Pearson correlation coefficient	
SL	Stride length	m
v	Walking velocity	m/s
W _Aqua_	Total external mechanical work in water per stride	J
W _D_	Horizontal work done against drag force per stride	J
W _v_	Vertical work done against apparent body weight per stride	J

### Self-selected speed in aquatic locomotion

**Fig. 6:**
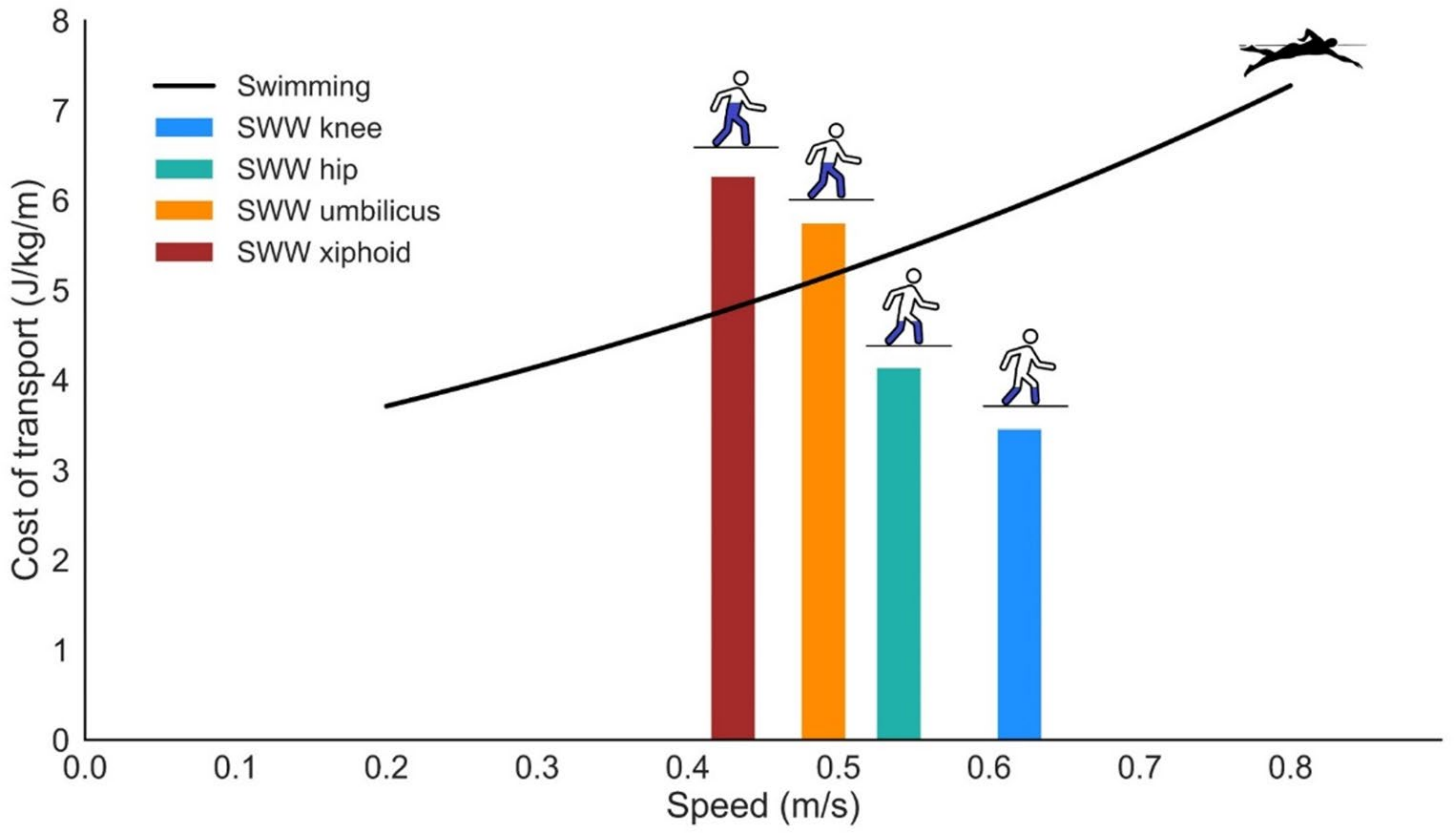
Cost of transport (COT, J/kg/m) during shallow water walking (SWW) at self-selected speeds compared to front crawl swimming. The colored bars represent the mean experimental COT during SWW at the self-selected speed for each immersion depth (knee: blue, hip: green, umbilicus: orange, xiphoid: brown). The black curve shows the predicted COT for crawl swimming as a function of speed, calculated using the equation from Capelli et al. [7]

The COT of SWW at self-selected walking speed was predicted using Eq. 6. As shown in Fig. 6, COT at self-selected walking speed increased with immersion depth (3.4 at knee, 4.1 at hip, 5.9 at umbilicus, 6.7 at xiphoid), whereas metabolic power (W/kg) at self-selected walking speed exhibit smaller absolute variation across depths (2.1, 2.2, 2.9, 2.9, respectively). For completeness, metabolic power at self-selected walking speed is also presented in alterenative units across the immersion depths (knee, hip, umbilicus, xiphoid): 6.3, 6.7, 8.6, 8.5 mlO_2_/kg/min; 30.4, 32.0, 41.2, 41.0 cal/kg/min; and 1.8, 1.9, 2.5, 2.5 metabolic equivalent of task (MET), respectively.

At self-selected walking speed, COT during SWW (Fig. 7) was higher than swimming at the xiphoid (0.43 m/s) and umbilicus (0.49 m/s) immersion depths. In contrast, COT at self-selected speed during SWW was lower than swimming at the hip (0.54 m/s) and knee (0.62 m/s) immersion depths. Moreover, self-selected walking speed during SWW decreased with increasing immersion depth and was lower at all depths compared to the self-selected swimming speed (1.11 m/s).

## Discussion

This study aimed to elucidate the mechanical determinants of the metabolic COT during SWW and compare its energetics to other forms of human locomotion. Our key findings provide insights into human aquatic movement.

Firstly, contrary to our initial hypothesis (H2) and the typical U-shaped curve observed in dry land walking, the COT during SWW did not exhibit a minimum at intermediate speeds, except for a J-shaped curve at knee immersion depth. Instead, the most economical condition occurred at the slowest speed (0.2 m/s) at hip immersion depth. This supports our first hypothesis (H1), suggesting that the interplay between the assistive effects of buoyancy (reducing vertical work against apparent weight) and the resistive effects of drag (increasing with speed and immersed area) determines the energetic cost. The minimum COT at hip immersion depth likely reflects an optimal balance, where buoyancy substantially reduces vertical work while drag has not yet become excessively high. Our physiomechanical model, which explicitly incorporated both buoyancy and drag forces, successfully predicted the measured COT, further corroborating the importance of this interplay.

Secondly, the comparison between SWW and swimming partly supported our third hypothesis (H3). SWW was metabolically more expensive than swimming only at deeper immersion depths (umbilicus and xiphoid) and higher speeds. This indicates the existence of an immersion depth threshold, likely near the body’s center of mass (between hip and umbilicus immersion depths), above which the increased frontal area makes walking against drag less economical than swimming. Notably, at self-selected speed, metabolic power remained relatively constant across immersion depths, whereas COT increased substantially with depth, suggesting that metabolic power may be a key regulated variable in self-selected speed SWW.

### Physiomechanical model of shallow water walking

Our physiomechanical model provides quantitative support for the hypothesis that the COT during SWW is primarily determined by the mechanical work against hydrostatic (buoyancy) and hydrodynamic (drag) forces. As shown in Fig. 2, increasing immersion depth leads to a rise in COT that mirrors the increase in drag force (DrF), occurring alongside a reduction in GRF_V_ due to buoyancy. This crucial role of drag is further highlighted by the gap between the COT surfaces (Fig. 3) of SWW developed with our experimental data and the dry land hypogravity walking extracted from Pavei and Minetti [31]. Since both conditions involve attenuated effective body weight, we can infer that the distance between the COT surfaces from each condition is greatly influenced by the hydrodynamic resistance of the water during SWW, particularly at greater immersion depths and walking speeds. Consequently, our physiomechanical model of SWW fundamentally differs from dry land hypogravity models (as from Pavei & Minetti [26]) by incorporating this essential hydrodynamic component alongside vertical forces.

To our knowledge, this study presents the first validated physiomechanical model that integrates both buoyancy and drag forces to predict the metabolic COT of human SWW. While previous SWW research has investigated various biomechanical and physiological variables [16], and sophisticated models exist for dry land hypogravity walking [9, 18, 19, 24, 27] or swimming energetics [39], none have provided a specific, validated framework linking the key aquatic forces to metabolic cost for the distinct mechanics of SWW. Although Orselli and Duarte [29] quantified drag forces and analyzed ground reaction forces during SWW, their study did not address metabolic cost. Our model, therefore, fills a critical gap, offering a novel tool for understanding human locomotion in aquatic environments. The validity of the physiomechanical model is supported by strong correlation between predicted and measured COT (R^2^ = 0.76), and further confirmed by robustness analyses. The LOOCV yielded a cross-validated R^2^ of 0.75, demonstrating the model’s strong ability to generalize and predict COT for unseen individuals, while the bootstrap analysis confirmed the stability of this correlation, yielding a 95% CI for R^2^ of [0.60–0.79] and indicating the strong relationship is unlikely due to chance.

The model and experimental data also shed light on optimization during SWW. Despite the general trend of increasing COT with immersion depth and walking speed, the minimum SWW COT occurred at hip depth and the slowest speed (0.2 m/s). This likely reflects a point of optimal balance where buoyancy reduces the vertical work needed to support body weight, while drag forces remain relatively low. Notably, the relationship between COT and speed deviated from the typical U-shaped seen on dry land. Only at knee immersion depth did the curve approximate a J-shaped (Fig. 6), with the minimum COT shifted towards the lowest speed. Pavei et al. [30] observed a maintained U-shaped curve during dry land hypogravity walking at 0.36 and 0.16 g (Mars and Moon gravities, respectively), whereas in SWW, this J-shaped pattern emerged exclusively at knee immersion depth (equivalent to 0.88 g). Although the other immersion depths had greater equivalent gravitational acceleration (0.58 g for hip, 0.48 g for umbilicus, 0.33 g for xiphoid) in comparison to the dry land hypogravity conditions from Pavei et al. [30], the COT curve at these deeper immersion depths always showed a constant positive slope. The drag force effect could account for this difference between SWW and dry land simulated hypogravity walking, generating important movement resistance, and increasing the COT steadily as the speed increases.

Finally, the comprehensive analyses indicated that the model is stable and responds predictably to variations in its input parameters (muscle efficiency, body mass, walking speed, and stride length). Furthermore, our finding of no significant difference in resting metabolic cost across immersion depths supports the interpretation that the observed COT differences during SWW are primarily driven by the mechanical effects of hydrodynamic and hydrostatic forces, rather than differential thermoregulatory demands.

#### Shallow water walking vs. swimming and dry land walking

Comparing the energetics of SWW with swimming and dry land walking provides a valuable context for understanding human adaptation to aquatic environments. We calculated iso-cost points – speeds at which different locomotor modes have similar metabolic energy requirements – to facilitate this comparison (Fig. 5; Table 3).

Our analysis revealed a critical dependence on immersion depth for the relative economy of SWW versus swimming. At shallower immersion depths (knee and hip), SWW consistently exhibited a lower COT than swimming across the tested speed range. However, at deeper immersion depths (umbilicus and xiphoid), a crossover occurred: SWW COT was more economical only at slower speeds, becoming metabolically more expensive than swimming at faster speeds. This finding supports our third hypothesis (H3) only under specific conditions and highlights that the most economical aquatic gait for humans is context-dependent. It suggests that at shallower immersion depths, the mechanical advantages associated with dry land walking, perhaps including some degree of pendular energy recovery [8], may persist, making SWW preferable. As immersion depth increases, the detrimental effects of drag likely become dominant, favoring the transition to swimming, particularly at higher speeds.

The existence of these iso-cost points between SWW and swimming only at deeper immersions suggests a potential energetic trigger for a gait transition, analogous to the walk-run transition on dry land [26]. Gait choice often represents a solution to an optimization problem aimed at minimizing metabolic cost [2]. Therefore, the transitioning from SWW to swimming at deeper immersion depths could be a behavioral adaptation to mitigate the rapidly increasing work against drag. Furthermore, our results suggest a potential immersion depth threshold for this transition near the body’s center of mass (between the hip and umbilicus immersion depths), where SWW become roughly as costly as swimming across much of the speed range (Fig. 5). The identification of this immersion depth threshold near the body’s center of mass influencing the most economic gait for human aquatic locomotion (SWW vs. swimming) is a novel finding in the literature, and it should be further investigated in the future.

Regarding optimization within SWW itself, the characteristic U-shaped COT curve seen in dry land walking [35] –representing metabolic optimization linked to mechanical parameters [2] – was roughly observed only at the shallowest immersion depth (a J-shaped curve at knee depth). This metabolic optimization typically occurs under conditions for which a species is evolutionarily adapted [38], like human walking on dry land [35]. Its presence only at knee immersion depth suggests that the form-function advantages of terrestrial locomotion may only partially persist at very shallow immersion depths. At deeper immersion depths, the optimization process appears overwhelmed by hydrodynamic forces, shifting the minimum COT toward the slowest speeds and altering the fundamental dynamics away from an effective pendular mechanism.

Finally, comparing SWW with dry land walking revealed that the iso-cost speed decreased as immersion depth increased. For example, SWW became as costly as dry land walking at 0.49 m/s for knee immersion depth but at only 0.25 m/s for xiphoid immersion depth. This inverse relationship is likely due to the higher drag force resistance experienced at deeper depths because of the greater body volume immersed, demanding more metabolic energy to walk.

### Self-selected speed in aquatic locomotion

We observed that the self-selected speed during SWW decreased as immersion depth increased (Fig. 6). This reduction likely stems from the greater drag force levels at deeper immersion depths. Building on Cavagna et al.’s [10] model for dry land walking, which attributes self-selected speed to an interplay between gravity and ground impact forces, we propose that drag acts as a third critical factor decelerating the body during SWW. This added resistance makes higher speeds increasingly difficult and less comfortable as more of the body becomes submerged.

Interestingly, despite the COT increases with immersion depth at these self-selected speeds, the metabolic power remained remarkably constant across all immersion depths. This suggests that the metabolic power, rather than COT, might be a key regulated parameter driving the choice of comfortable walking speed in water. As drag increases with immersion depth, individuals appear to compensate by slowing down to maintain a preferred, near-constant rate of energy expenditure. This near-constant metabolic power (ranging from 1.8 METs at the knee to 2.5 METs at the xiphoid) has practical implications, suggesting SWW offers a predictable level of energy expenditure for physical activity, regardless of minor variations in immersion depth when walking at a comfortable pace.

Comparing self-selected speed SWW to swimming reinforces the concept of an immersion depth threshold. At the self-selected speeds, SWW was more economical than swimming only at shallower immersion depths (knee and hip) (Fig. 6). This observation supports the idea that the biomechanical advantages inherent to human terrestrial walking are only beneficial below a certain immersion depth. The substantial increase in self-selected speed increases when transitioning from walking to swimming (approximately twofold) further suggests that changing gait is an optimization strategy employed by humans to move more effectively in deeper water.

#### Limitations and perspectives

Our study has some limitations that warrant consideration. It focused solely on mechanical work, reflecting the net effect of all muscle-tendon units and joint structures. We did not account for force sharing among synergist muscles, co-contraction between antagonists, or energy transfer between segments. Training adaptations may also modify limb mechanics, potentially influencing transition speeds. Comparisons with swimming COT should be made carefully, as the equation used to compute the swimming COT was extrapolated from a study [7] of a distinct range of speed. Additionally, because the walking speed during SWW was controlled by auditory cues, participants deviated from the target speed only in the fastest walking speed condition of 0.8 m/s, as indicated by the results of simple t-tests; however, this deviation is unlikely to affect the overall interpretations of the findings.

The present results provide insights for therapists, coaches, and researchers by contributing a physiomechanical perspective to the dose-response of shallow water walking as an aquatic therapy. Manipulating speed and immersion depth can help optimize therapeutic outcomes across different movement disorders. Future research should extend these analyses to other populations –such as females, older individuals, painful conditions, neuromuscular disorders - to better understand the interaction between the mechanical forces and energy expenditure during SWW. Longitudinal studies are also encouraged to examine how exercise interventions influence these physiomechanical parameters over time.

## Conclusion

In this study, we investigated the physiomechanical determinants of the COT during SWW in humans. We quantified COT alongside buoyancy and drag force across four speeds and four immersion depths, developed and validated a novel physiomechanical model integrating these forces, and compared the energetics of SWW with swimming.

Our findings indicated that the minimum COT for SWW occurred at hip immersion depth and the slowest speed (0.2 m/s), suggesting an optimal balance in which buoyancy reduces vertical work without excessive drag force resistance. Furthermore, our comparative analysis revealed an immersion depth threshold near the body’s center of mass: below this threshold, SWW at self-selected speeds was more economical than swimming, whereas above it, swimming became advantageous. Notably, metabolic power remained relatively constant across immersion depths during self-selected walking, suggesting it may be a key regulated variable influencing preferred speed in water.

Collectively, these results highlighted the critical interplay between buoyancy and drag in shaping the energetics of human aquatic locomotion and identified an immersion depth threshold influencing the choice between walking and swimming.

## Supplementary Information

Below is the link to the electronic supplementary material.

Supplementary Material 1

Supplementary Material 2

## Data Availability

The individual dataset is available on-line (doi: 10.6084/m9.figshare.13221485) and in Supplementary Material 2.

## References

[1] Alexander RM (1989) Optimization and gaits in the locomotion of vertebrates. Physiol Rev 69:1199–1227. 10.1152/physrev.1989.69.4.1199 2678167 10.1152/physrev.1989.69.4.1199

[2] Alexander RMN (2006) Principles of animal locomotion. Princeton University Press, Princeton

[3] Ardigò LP, Saibene F, Minetti AE (2003) The optimal locomotion on gradients: Walking, running or cycling? Eur J Appl Physiol 90:365–371. 10.1007/s00421-003-0882-7 12898263 10.1007/s00421-003-0882-7

[4] Barela AMF, Stolf SF, Duarte M (2006) Biomechanical characteristics of adults walking in shallow water and on land. J Electromyogr Kinesiol 16:250–256. 10.1016/j.jelekin.2005.06.013 16111894 10.1016/j.jelekin.2005.06.013

[5] Baron B, Dekerle J, Depretz S, Lefevre T, Pelayo P (2005) Self selected speed and maximal lactate steady state speed in swimming. J Sports Med Phys Fitness 45:1–6 16208283

[6] Borg G (1990) Psychophysical scaling with applications in physical work and the perception of exertion. Scand J Work Environ Health 16:55–58. 10.5271/sjweh.1815 2345867 10.5271/sjweh.1815

[7] Capelli C, Pendergast DR, Termin B (1998) Energetics of swimming at maximal speeds in humans. Eur J Appl Physiol Occup Physiol 78:385–393. 10.1007/s004210050435 9809837 10.1007/s004210050435

[8] Cavagna GA, Thys H, Zamboni A (1976) The sources of external work in level walking and running. J Physiol 262:639–657. 10.1113/jphysiol.1976.sp011613 1011078 10.1113/jphysiol.1976.sp011613 PMC1307665

[9] Cavagna GA, Willems PA, Heglund NC (1998) Walking on Mars. Nature 393:636. 10.1038/31374 9641676 10.1038/31374

[10] Cavagna GA, Willems PA, Heglund NC (2000) The role of gravity in human walking: pendular energy exchange, external work and optimal speed. J Physiol 528:657–669 11060138 10.1111/j.1469-7793.2000.00657.x PMC2270143

[11] Gabrielsen A, Johansen LB, Norsk P (1993) Central cardiovascular pressures during graded water immersion in humans. J Appl Physiol 75:581–585. 10.1152/jappl.1993.75.2.581 8226455 10.1152/jappl.1993.75.2.581

[12] GilbertW Gleim, Nicholas JA (1989) Metabolic costs and heart rate responses to treadmill walking in water at different depths and temperatures. Am J Sports Med 17:248–252. 10.1177/036354658901700216 2757128 10.1177/036354658901700216

[13] Gobbo S, Bullo V, Duregon F, Cugusi L, Vendramin B, Bocalini DS, Benelli P, Alberton CL, Di Blasio A, Cruz-Diaz D, Bergamo M, Ermolao A, Bergamin M (2017) A comparative analysis between head-out underwater walking and land-based treadmill walking in a group of healthy asymptomatic elderly. Sport Sci Health 13:583–589. 10.1007/s11332-017-0387-0

[14] Harrison R, Bulstrode S (1987) Percentage weight-bearing during partial immersion in the hydrotherapy pool. Physiotherapy Practice 3:60–63. 10.3109/09593988709087741

[15] Holmér I (1972) Oxygen uptake during swimming in man. J Appl Physiol 33:502–509. 10.1152/jappl.1972.33.4.502 5075849 10.1152/jappl.1972.33.4.502

[16] Ivaniski-Mello A, Zimmermann Casal M, Costa RR, Alberton CL, Martinez FG, Peyré-Tartaruga LA (2023) Quantifying physiological and biomechanical responses of shallow water walking: a systematic review and meta-analysis. Res Sports Med 31:604–618. 10.1080/15438627.2021.2020786 34979836 10.1080/15438627.2021.2020786

[17] Kipp S, Byrnes WC, Kram R (2018) Calculating metabolic energy expenditure across a wide range of exercise intensities: the equation matters. Appl Physiol Nutr Metab 43:639–642. 10.1139/apnm-2017-0781 29401411 10.1139/apnm-2017-0781

[18] Kluis L, Wynn C, Kennedy D, Diaz-Artiles A (2025) Characterization of the effects of sex, speed, and incline on metabolic rate during partial gravity ambulation. J Appl Physiol 139:787–796. 10.1152/japplphysiol.00627.2024 40879065 10.1152/japplphysiol.00627.2024

[19] Kraft JC, Augustine JA, Fiddler RE, Lewis C, Dames KD (2023) Bodyweight support alters the relationship between preferred walking speed and cost of transport. Hum Mov Sci 88:103068. 10.1016/j.humov.2023.103068 36806975 10.1016/j.humov.2023.103068

[20] Kruel LFM (1994) Peso hidrostático e frequência cardíaca em pessoas submetidas a diferentes profundidades de água. Universidade Federal de Santa Maria

[21] Kuliukas AV, Milne N, Fournier P (2009) The relative cost of bent-hip bent-knee walking is reduced in water. Homo 60:479–488. 10.1016/j.jchb.2009.09.002 19853850 10.1016/j.jchb.2009.09.002

[22] Lindsey CC (1978) Form, function, and locomotory habits in fish. In: Soar WS, Randall DJ (eds) Fish Physiology. Academic Press, pp 1–100

[23] Louder TJ, Searle CJ, Bressel E (2016) Mechanical parameters and flight phase characteristics in aquatic plyometric jumping. Sports Biomech 15:342–356. 10.1080/14763141.2016.1162840 27125295 10.1080/14763141.2016.1162840

[24] MacLean MK, Ferris DP (2022) Effects of simulated reduced gravity and walking speed on ankle, knee, and hip quasi-stiffness in overground walking. PLoS One 17:e0271927. 10.1371/journal.pone.0271927 35944021 10.1371/journal.pone.0271927 PMC9362947

[25] Minetti AE (1998) The biomechanics of skipping gaits: a third locomotion paradigm? Proc Biol Sci 265:1227–1235. 10.1098/rspb.1998.0424 9699315 10.1098/rspb.1998.0424 PMC1689187

[26] Minetti AE, Ardigò LP, Saibene F (1994) The transition between walking and running in humans: metabolic and mechanical aspects at different gradients. Acta Physiol Scand 150:315–323. 10.1111/j.1748-1716.1994.tb09692.x 8010138 10.1111/j.1748-1716.1994.tb09692.x

[27] Minetti AE, Luciano F, Natalucci V, Pavei G (2024) Horizontal running inside circular walls of Moon settlements: a comprehensive countermeasure for low-gravity deconditioning? R Soc Open Sci 11. 10.1098/rsos.231906 10.1098/rsos.231906 PMC11076109 38716331

[28] Newman DJ (1992) Human Locomotion and Energetics. Massachusetts Institute of Technology

[29] Orselli MIV, Duarte M (2011) Joint forces and torques when walking in shallow water. J Biomech 44:1170–1175. 10.1016/j.jbiomech.2011.01.017 21334630 10.1016/j.jbiomech.2011.01.017

[30] Pavei G, Biancardi CM, Minetti AE (2015) Skipping vs. running as the bipedal gait of choice in hypogravity. J Appl Physiol 119:93–100. 10.1152/japplphysiol.01021.2014 25930029 10.1152/japplphysiol.01021.2014

[31] Pavei G, Minetti AE (2016) Hopping locomotion at different gravity: metabolism and mechanics in humans. J Appl Physiol 120:1223–1229. 10.1152/japplphysiol.00839.2015 26635350 10.1152/japplphysiol.00839.2015

[32] Péronnet F, Massicotte D (1991) Table of nonprotein respiratory quotient: An update. Can J Sport Sci 16:23–29 1645211

[33] Peyré-Tartaruga LA, Coertjens M (2018) Locomotion as a powerful model to study integrative physiology: efficiency, economy, and power relationship. Front Physiol 9:1–16. 10.3389/fphys.2018.01789 30618802 10.3389/fphys.2018.01789 PMC6297284

[34] Peyré-Tartaruga LA, Dewolf AH, di Prampero PE, Fábrica G, Malatesta D, Minetti AE, Monte A, Pavei G, Silva-Pereyra V, Willems PA, Zamparo P (2021) Mechanical work as a (key) determinant of energy cost in human locomotion: recent findings and future directions. Exp Physiol 106:1897–1908. 10.1113/EP089313 34197674 10.1113/EP089313

[35] di Prampero PE (1986) The energy cost of human locomotion on land and in water. Int J Sports Med 7:55–72 3519480 10.1055/s-2008-1025736

[36] Saibene F, Minetti AE (2003) Biomechanical and physiological aspects of legged locomotion in humans. Eur J Appl Physiol 88:297–316. 10.1007/s00421-002-0654-9 12527959 10.1007/s00421-002-0654-9

[37] Schmidt-Nielsen K (1972) Locomotion: Energy cost of swimming, flying, and running. Science 21:222–228. 10.1126/science.177.4045.222 10.1126/science.177.4045.222 4557340

[38] Toussaint HM, Stralen M van, Stevens E (2002) Wave drag in front crawl swimming. 20 International Symposium on Biomechanics in Sports 279–282

[39] Weibel ER (2000) Symmorphosis: On form and function in shaping life. Harvard University Press, London, England

[40] Zamparo P, Cortesi M, Gatta G (2020) The energy cost of swimming and its determinants. Eur J Appl Physiol 120:41–66. 10.1007/s00421-019-04270-y 31807901 10.1007/s00421-019-04270-y

