# Supplementary material for: An in-depth look at shallow-water walking: the mechanical determinants of the energy metabolic cost of shallow water walking in humans
Source: Pflugers Arch. 2025 Dec 12;478(1):7. doi: 10.1007/s00424-025-03130-3 (PMC12698785; doi:10.1007/s00424-025-03130-3)
Supplement: Supplementary file 1 — Supplementary Material 1 [file 424_2025_3130_MOESM1_ESM.docx]

**Pflügers Archiv - European Journal of Physiology**

**An in-depth look at shallow-water walking: The mechanical determinants of the energy metabolic cost of shallow water walking in humans**

**André Ivaniski-Mello^1^, Alberto Enrico Minetti^2^, Flávia Gomes Martinez^1^, Leonardo Alexandre Peyré-Tartaruga^3, 4^***

*^1^ LaBiodin Biodynamics Laboratory, Universidade Federal do Rio Grande do Sul, Porto Alegre, Brazil*

*^2^ Laboratory of Physiomechanics of Locomotion, Department of Pathophysiology and Transplantation, Physiology Division, University of Milan, Via Mangiagalli 32, 20133, Milan, Italy*

*^3^ Human Locomotion Laboratory (LocoLab), Department of Public Health, Experimental and Forensic Medicine, University of Pavia, Pavia, Italy*

*^4^ Departamento de Biofísica, Facultad de Medicina, Universidad de la República, Montevideo, Uruguay*

*** Corresponding author:** Leonardo A. Peyré-Tartaruga, Human Locomotion Laboratory (LocoLab), Department of Public Health, Experimental and Forensic Medicine, University of Pavia, Via Forlanini 2, 27100, Pavia, Italy - E-mail: leonardo.tartaruga@unipv.it

**Supplementary Material 1**

**Physiomechanical model of human shallow water walking**

1. **Forces estimation**

We estimated the hydrodynamic and hydrostatic forces acting on the immersed body during the stride cycle of shallow water walking. The hydrodynamic force is the Drag Force (DrF) resisting the body displacement along the water. The hydrostatic force is the buoyancy force, attenuating the downward acceleration due to gravity force, therefore reducing the apparent body weight when immersed.

***Drag Force***

The DrF is composed by pressure DrF, frictional DrF, and wave DrF [5]. Nevertheless, Newman [3] found that the only the pressure DrF seems to be of an important magnitude during human shallow water walking (Reynold number values between 0.82 x 10^5^ and 6.88 x 10^5^); thus in the present study we assumed the pressure DrF as the only hydrodynamic resistance applied by the fluid on the moving body.

The DrF (N) resistance suffered by the immersed body during the stride cycle was estimated by the mathematical model proposed by Orselli & Duarte [4] - the reader can obtain more details about the model in the cited reference, but here we give a brief explanation. This model considers the fluid interaction as a stationary flow, ignores non-inertial effects such as added-mass terms, and does not take into account the friction between the skin and water.

The DrF was considered as

$\vec{DrF}=-\frac{C_{d}\rho_{m}A_{\perp}v^{2}}{2}\hat{v}$ (1)

Where $C_{d}$ is the drag coefficient, $\rho_{m}$ (kg/m^3^) is the water density, $A_{\perp}$ (m) is the projection of the frontal area in a plane perpendicular to the segment velocity, $v^{2}$ (m/s) is the square of the segment velocity, and $\hat{v}$ is a unitary vector in the direction of the segment velocity.

The $C_{d}$ value adopted was 1, and $\rho_{m}$ was considered as 1000 kg/m^3^. The $A_{\perp}$ was determined by a geometrical modeling of each immersed body segment. The $v^{2}$ was calculated with kinematic data, and $\hat{v}$ was considered the vector contained in the sagital plane pointing in the antero-posterior direction parallel to the floor.

The immersed body segments were modelled as geometrical solids and their dimensions were calculated from 20 anthropometric measurements obtained from the trunk, thigh, shank, and foot of each subject. The foot was modeled as an elliptical solid with a circular base, the leg as a truncated circular cone, the thigh as an elliptical solid with a circular top, the lower trunk as an elliptical column, and the mid trunk as an elliptical solid.

During the stride cycle, the body segments perform both movements of translation and rotation, leading to a gradient of velocities for the different parts of each body segment. This unequal velocity magnitude along the body segment parts leads to distinct DrF acting on each body segment part. To estimate the total DrF acting on the body segment, each segment was first divided into several thin strips, the DrF (Eqn. 2) resisting on each strip was calculated, and then the total DrF of the segment was determined by the integration of the DrF from the individual strips (Eqn. 3).

$\vec{dDrF}= -\frac{C_{d}\rho_{m}{dA}_{\perp} v^{2}}{2}\hat{v}$ (2)

$\vec{DrF}=\int_{0}^{L} \vec{dDrF}dz$ (3)

Where $\vec{dDrF}$ is the infinitesimal DrF acting on each strip of ${dA}_{\perp}$ frontal projected area, $dz$ is the differential along the longitudinal axis $z$ from the body segment, and $L$ (m) is the segment length.

The DrF was estimated for each point in time in the entire stride cycle, and the DrF of each immersed body segment was integrated for all stride cycle. The total DrF were determined by the summation of all body segments DrF.

***Buoyancy force***

The buoyancy force effect was estimated by its weight attenuation effect during the immersion. To estimate the apparent body weight in percentage from dry land weight, we used the data from Kruel [1] of weight reduction during immersion in the depths of knee, hip, umbilical and xiphoid levels. Since during the stride cycle the mean vertical ground reaction force (GRF_v_) (N) is equal to the body weight [2], we estimated the apparent body weight of each participant in each immersion depth and considered this weight as the stride cycle GRF_v_.

**References**

1. Kruel LFM (1994) Peso hidrostático e frequência cardíaca em pessoas submetidas a diferentes profundidades de água. Universidade Federal de Santa Maria

2. Minetti AE (1998) The biomechanics of skipping gaits: a third locomotion paradigm? Proceedings of the Royal Society B: Biological Sciences 265:1227–1235. doi: 10.1098/rspb.1998.0424

3. Newman DJ (1992) Human Locomotion and Energetics. Massachusetts Institute of Technology

4. Orselli MIV, Duarte M (2011) Joint forces and torques when walking in shallow water. J Biomech 44:1170–1175. doi: 10.1016/j.jbiomech.2011.01.017

5. Toussaint HM, Stralen M van, Stevens E (2002) Wave drag in front crawl swimming. 20 International Symposium on Biomechanics in Sports 279–282
